# Boston Letter

**Published:** 1881-05

**Authors:** 


### Domestic Correspondence.

---

### ARTICLE XII.

### BOSTON LETTER.

MESSRS. EDITORS:—In my February letter I mentioned a discussion upon the paper of Dr. James H. Whittemore, Resident Physician of the Massachusetts General Hospital, upon the abuse of the medical charities of Boston. The outcome of the matter is a circular recently issued by order of the trustees of this hospital, which wisely sets forth that "On and after the first of April the out-patient medical and surgical service will be restricted to such sick and disabled poor persons as are unable to pay fees. No others will be treated." The circular further states that this restriction has been compelled by an increasing attendance in this department, which already is overcrowding the resources of the hospital, and if allowed to continue will soon become unmanageable. During the year 1880 more than 37,000 visits were recorded. It has been discovered that many of these patients were not in need of charitable treatment. They quite inconsistently come long distances at considerable expense, and interfere with patients who are actually too poor to pay either fees or fares. This is a species of facial sclerosis which will no longer be tolerated. The medical attendance under the administration of this department of the hospital, has more than doubled within a single decade. The trustees appreciate the embarrassing condition of things, and while they will take firm stand hereafter in protecting the interests of the deserving poor, the limited funds of the hospital, and the proper management of their out-patient department, they will at the same time give a lesson in self-respect to the parasites who naturally will try to continue their robbery of the hospital and of the outside physicians, to whom their cases, and the fees they should pay, fairly belong. Certainly this vigorous action on the part of the trustees should serve as an example in other cities as well as in our other medical charities. At first there will probably be some difficulty in separating the sheep from the goats, but news of this nature usually flies swiftly, and we may expect to see the number of hypocrites rapidly diminish.

I suppose that no class of workers in this busy world is so much abused and so little appreciated as physicians. We all know that but comparatively few persons find any pleasure in paying a doctor's bill. "Gratitude in a patient," says a quaint writer, "is a symptom of the disease, and it steadily lessens as the patient grows better." The doctor needs the fee for which he has given his time, experience and knowledge, but I trust he has a higher object in view, and if he relieves his patient, the satisfaction he thus derives should certainly be of greater value to him than mere money. If at the same time he receive an expression of grateful appreciation from his patient, he feels that the anxiety and care which the case has cost him have met a due reward. But the coolness with which many call for the physician's advice, without the slightest intention of making any return for it, and the settled belief in their warped brains, that the doctor's counsel costs him nothing, and that therefore he might just as well *give* it as *sell* it, indicate a moral strabismus which is past the understanding of all but the crookedest of pocket-picking human beings.

To turn to a more congenial topic: We have a Hospital Newspaper Society which is doing a most kindly and meritorious work. From boxes in the railroad stations, during the past year, over eighty-three thousand newspapers have been collected for daily distribution in the Massachusetts general and city hospitals. Sixty-three hundred magazines and books have been sent to various insane asylums, reformatory schools and lighthouses. Three thousand Christmas cards were sent to the insane, much to their delight. The report of the society shows that not only light reading, but books and magazines of solid character, have been equally appreciated. One poor fellow in an insane asylum has been kept quiet and happy by an *algebra* ever since he received it. Hardly anything in the shape of reading matter comes amiss, and much more could be used.

If the king who entertained his horses by means of orchestral concerts lived in our day, he doubtless would insist upon giving the patients of his hospitals something better than mere bare, white walls for mental recreation. There must be a vast amount of depression among the sick in our public institutions, which cannot be relieved by a daily visit and *ter in die* doses of hospital compounds. If the minds of these people could be enlivened by cheering mental food of any nature, who doubts that the average death-rate would be lessened and the average duration of illness be shortened? The *London Spectator* has lately advocated the introduction into hospitals not only of flowers and plants, but of paintings, statuary and other works of art and beauty. It is a wise, a very wise suggestion. "Patients will tell you," says the *Spectator*, "with something like enthusiasm, of their joy as they heard the swish of the swaying, wind-tossed trees under their chamber windows, or watched the twinkling motion of the leaves of a tall poplar, the top of which might be seen from their beds. They never forget how they chanced in their many waking hours to see the moon hovering over a neighboring spire—like the dot to an i, as Alfred de Musset has it. A chance flower, a bit of verse repeated by a nurse, a scrap of music wafted from the street below, a pretty sentence in the letter of a friend, will thrill them as they never were moved before, and will cling, limpet-like, to their memories. 'I shall never forget,' says one, 'the rapture of fever patients over a bunch of bright-colored flowers, and the sight of the baby was sometimes more to patients than the visit of the doctor.' Nurses will tell you that there often comes to the most callous and withered human beings, in these times, a restless longing to see something fresh and beautiful, and that the most obtuse betray a craving to look out of the window. These are the times when the parched mind, barely capable of bearing the cheap, common grain of life, seems to show possibilities of fruit and flower; and why not use the opportunity?" The *Spectator* thinks the development of art in hospitals would be beneficial to artists as well as to the sick. For it would practically do away with the complaint among the best and most earnest-minded artists, that they so often have to paint for rather frivolous objects, quite unworthy of their capabilities. Their best pictures would no longer be buried in private houses, but would be the means of achieving great good. This opens to the charitable among the wealthy an opportunity to devote their money to a most useful and noble purpose. Let them ornament our hospitals with good and influential pictures, whether paintings or engravings. In some of the London hospitals there are pictures which not only break the monotony of the customary blank and cheerless walls, but they act most pleasantly upon the bed-ridden unfortunates who have them in view. Why not encourage this species of charity and good-feeling in our own country ?

Art in connection with clinical teaching has also been suggested of late. In a lecture upon the anatomy of the joints, Dr. W. W. Keen, of Philadelphia, and Professor of Artistic Anatomy in the Pennsylvania Academy of the Fine Arts, made use of a living model to illustrate and demonstrate his subject. It was then that he affirmed that the living model should form as indispensable a part of the means of illustration in the anatomical lecture room as the cadaver. When we think of what uses might be made of such a model, it becomes very apparent that Dr. Keen's suggestion is one which deserves practical adoption.

The new medical college to which I have alluded in a former letter, is a curious puzzle to the general medical mind of Boston. The common hope is that it will do no harm. The fact that its advertisement is admitted to the pages of the *Boston Medical and Surgical Journal* proves nothing more than that this is probably a quieter method of disposing of it than its rejection. One of the professors of the school applied for admittance to our State society. During his examination, he stated that his dose of the potassium iodide was one to two drachms, but added that he did not use it in his practice. This proves whatever you think best.

Before our State legislature is a bill " to prevent incompetent persons from conducting the business of apothecaries." The requirements of the bill are quite stringent, and it is hoped it may pass. We do not forget, however, the ease with which the bill for the regulation of the practice of medicine was defeated last winter, and therefore are not over-sanguine in our hopes of the good fate of the pharmacy bill. It is a matter for sincere rejoicing that in spite of the freedom with which Tom, Dick and Harry are allowed to compound prescriptions and do counter-practice, there are comparatively so few ill results. But the dangers of illegitimate and ignorant pharmacy are immense, greater probably than those of quackery. May the day soon come when our country will be protected by medical and pharmaceutical laws as strict as those now in force in Germany.

BOSTON, April, 1881. * *

---

PATHOGNOMONIC SIGN OF FRACTURE OF THE NECK OF THE FEMUR.—Dr. Bezzi draws attention, in *Lo Spallanzani*, to a sign which is pathognomonic of fracture of the neck of the femur, but which is not generally known (*Buffalo Medical and Surgical Journal*). In examining the space between the trochanter and the crista ilii, it will be found that while, on the sound side, the muscles occupying this region (the tensor vaginæ femoris and the gluteus medius) are tense, and offer to the hand a considerable feeling of resistance, they present on the affected side a deep, well marked depression, a flaccidity, and diminution of tension, from displacement upward of their points of insertion.

THE INFLUENCE OF INJURIES AND SURGICAL OPERATIONS ON HEREDITARY DESCENT.—" M. Mason, of the Royal Academy of Belgium, has experimentally demonstrated that the removal of the spleen in the parent will insure a rudimentary state of that organ in the offspring. The animals experimented upon were rabbits, and the atrophic spleen of the descendant of an operated animal weighed only 9.32, the bodily weight being 1.472 grains. In the second generation of these descendants the atrophy was not more pronounced than in the first."—*Chicago Medical Review, March* 20.

